# A short review on the role of Artificial Intelligence (AI) in dental implants

**DOI:** 10.6026/973206300211824

**Published:** 2025-07-31

**Authors:** Rahul K. Chaudhari, Srilatha T., Vipin Dehane, Chinmay Gokhale, Pragathi R. Bhat, Hitesh Desarda

**Affiliations:** 1 Department of Oral and Maxillofacial Surgery, Government Dental College and Hospital, Jalgaon, Maharashtra, India; 2 Consultant, Oral and Maxillofacial Surgery, Hyderabad, India; 3 Department of Oral and Maxillofacial Surgery and Department of Dentistry and Maxillofacial Surgery, Fortis Hospital, Mumbai, Maharashtra, India; 4 Department of Oral and Maxillofacial Surgery, YCM Dental College, Ahilyanagar, Maharashtra, India; 5 Department of Periodontics and Oral Implantology, SDM College of Dental Sciences and Hospital, Dharwad, Karnataka, India; 6 Department of Periodontics, Government Dental College and Hospital, Jalgaon, Maharashtra, India

**Keywords:** Artificial intelligence, Dental Implant, Implant dentistry, robotic surgery, virtual reality

## Abstract

Implant dentistry heavily depends on Artificial Intelligence (AI) technology. This will modify the current state of medical practices using scientific evidence. The integration of AI in implant dentistry has emerged as a transformative approach, enhancing precision, efficiency, and predictability across various stages of treatment. AI's potential in diagnosis, treatment planning, surgical execution is the game changer in implant dentistry. Hence, we the known potential of AI to redefine clinical work and improve treatment outcomes in implant dentistry.

## Background:

The term "Artificial Intelligence" was introduced by John McCarthy in 1955 [1]. Artificial intelligence (AI) has revolutionized many fields in healthcare including dentistry, and specifically implant dentistry; a field that demands precision, careful planning, and accurate execution. Implant dentistry, which involves replacing missing teeth with artificial implants. Traditional methods depend on the clinician's expertise, which can vary. In contrast, AI uses data from imaging systems like cone-beam computed tomography (CBCT) scans and panoramic radiographs to process and interpret diagnostic images with unparalleled precision. AI can detect key anatomical structures such as the inferior alveolar nerve and maxillary sinus etc. reducing human errors [2]. In addition, AI algorithms provides clear picture of bone quality and volume, helping clinicians to determine implant feasibility and areas for bone augmentation. AI automates implant placement with exact angulation which varies from case to case. This streamlines the planning process and allows clinicians to visualize treatment outcomes in advance. AI-enhanced robotic systems provide real-time feedback during surgery, enabling clinicians to place implants with exceptional accuracy [3]. These robots follow pre-determined plans and adjust based on dynamic inputs during the procedure, which is especially helpful in complex cases. In addition augmented reality (AR) develops the digital treatment plan based on the patient's anatomy, guiding clinicians during implant placement. AI facilitates earlier disease detection, perfect treatment plan, improved patient care and perfect treatment outcome. Moreover, AI also identifies early warning signs of complications such as peri-implantitis, allowing timely interventions [4]. Mobile applications and wearable sensors enable continuous monitoring of implant stability and oral health. The role of Artificial Intelligence (AI) in implant dentistry is outlined [11]. Further, the applications of artificial intelligence in dental implant planning are also known [12]. Therefore, it is of interest to explore AI's current role in implant dentistry, its impact on practice, and the future of AI-driven dental care.

## AI-based patient management:

AI accurately predicts treatment outcomes and potential complications using patient history and data analysis. It streamlines the documentation process to maintain accurate and up-to-date patient records, while AI Chatbots enhance patient communication by providing information and follow-up care instructions to improve patient engagement and education [5]. This integrated approach ensures personalized care, enabling healthcare providers to focus more on clinical tasks while reducing administrative burdens. Ultimately, such advancements lead to improved patient satisfaction and more efficient healthcare delivery.

## Discussion:

AI completely changed the implant dentistry by Assessing in diagnosis, Treatment planning, by detection of Implant type or brand, accurate execution of implant surgery and Predicting accurate treatment outcome. AI-driven technologies like robotic systems and dynamic navigation tools reduce human errors and ensuring precise implant placement [5]. These systems offer real-time guidance, providing immediate feedback to clinicians and helping them maintain the planned trajectory during surgery. Cone beam CT (CBCT) scans are the gold standard for dental implant planning. It is very hard for general dental practitioners to evaluate CBCT scans for detailed implant planning and identification of anatomical structures. AI can contribute to solving this problem upto the acceptable accuracy. The measurement of bone height and width for implant placement by AI has limited success, research states that AI can serve as a valuable tool for the detection of anatomical landmarks. Morgan *et al.* stated that AI model provides consistent automatic segmentation of maxillary sinus, which allowed precise reproduction of 3D models for diagnosis and treatment planning [6]. Furthermore AI can be used to predict the primary stability of implants based on the drilling protocols pre-surgery. This could be of great help to young clinicians who are at the start of their implantology careers [7]. AI also improves the design of surgical guides by accurately mapping patient anatomy, which enhances implant stability and reduces the risk of misplacement. AI allows for personalized care by analysing patient-specific data such as bone density, occlusal forces, and oral health, creating customized treatment plans. It also predicts potential complications based on individual risk profiles, enabling clinicians to implement preventive measures, such as pre-surgical bone augmentation. AI-integrated CAD/CAM systems improve prosthetic designs, matching them to the patient's dental arch for better functionality and aesthetics [8]. In case of any complications with the implants or their super-structures it may be difficult or even impossible to get the information regarding implant manufacturer, diameter, length, platform, and abutment type etc. if the procedures have been performed at another clinic. In this scenario The CNNs model of AI can identify images by forming an identification algorithm in which they can detect the spatial hierarchies of features, such as edges, textures, shapes and helps in identification of implant brands [8]. Accuracy and feasibility of the detection of different dental implant systems (DIS) by AI is now emerging. As dental implants are the most preferred treatment modality, implant complications are also on the rise. Prediction of treatment outcomes in implantology is the need of the hour, and AI has the potential to be a major contributor to this field. There are no systematic reviews and meta-analyses yet published on the prediction of treatment outcomes. Lyakhov *et al.* proposed a neural network model for predicting survival rates of single dental implants by analysing the statistical factors of the patients [9]. Huang *et al.* suggested that their predictive AI model using neural networks can suggest the implant fate within five years, which will help dentists identify high-risk patients and accordingly modify their treatment plans [10]. The integration of robotics and AI in dentistry is called "Dentronics". A case was performed in China in 2017 where two implants were placed in a patient by a robot. Several clinical reports were published in the literature where successful implant placements have been done by robots [10].

## Conclusion:

Integrating AI in implant dentistry offers transformative potential, enhancing diagnosis, treatment planning, surgical precisions and post-operative care. However, AI in implant dentistry faces challenges, including data dependency, limited access to standardized dental datasets, substantial investment in infrastructure and clinician training, which can limit its adoption, particularly in resource-limited settings.

## Financial support and sponsorship:

Nil.
